# Impact of preoperative delay on perforation in pediatric appendicitis

**DOI:** 10.3389/fped.2026.1850522

**Published:** 2026-08-04

**Authors:** Lixiang Meng, Yongpeng Duan, Decheng Wei, Zhongce Li, Zhikun Zhang, Shiqin Qi

**Affiliations:** Department of General Surgery, Anhui Provincial Children’s Hospital, Hefei, China

**Keywords:** acute appendicitis, appendiceal fecalith, appendiceal perforation, children, laparoscopic appendectomy, preoperative delay, uncomplicated acute appendicitis

## Abstract

**Objective:**

To investigate the impact of preoperative delay (<8 h vs. <24 h) on appendiceal perforation in children with uncomplicated acute appendicitis (UAA) undergoing laparoscopic appendectomy, and to assess the risk associated with appendiceal fecalith.

**Methods:**

A retrospective study included children with UAA who underwent laparoscopic appendectomy from January 2025 to December 2025. Based on preoperative waiting time, children were divided into control group (<8 h, *n* = 122) and observation group (<24 h, *n* = 75). Primary outcome was intraoperative perforation rate (AAST grade 3–5). Secondary outcomes included operation time, hospital stay, pathological results, complications, and costs.

**Results:**

Baseline data were comparable between groups (*P* > 0.05). The AAST perforation rate was 7.38% (9/122) in the control group and 9.33% (7/75) in the observation group (*P* > 0.05). No significant differences were found in operation time, hospital stay, complications, or costs (*P* > 0.05). In the fecalith subgroup, no significant difference in AAST perforation rates was observed (*P* > 0.05), but significant differences were found in operation time [38.7 vs. 41.0 min, *P* = 0.045], pathological perforation rate (12.16% vs. 28.21%, *P* = 0.034), and preoperative delay time (6.21 ± 2.00 vs. 13.89 ± 2.23 h, *P* < 0.001). Appendiceal fecalith was identified as a risk factor for perforation in delayed surgery. No serious complications occurred during 30-day follow-up.

**Conclusion:**

Delaying laparoscopic appendectomy to within 24 h for children with UAA does not significantly increase the risk of intraoperative perforation compared to surgery within 8 h. Such surgeries can be safely scheduled for the next morning to optimize night shift resource allocation. However, children with preoperative evidence of appendiceal fecalith are at higher risk of perforation and should receive timely surgical intervention.

## Introduction

Acute appendicitis is one of the most common acute abdominal conditions, and laparoscopic appendectomy (LA) is currently the standard treatment. Traditional belief holds that, once diagnosed, the appendix should be removed as soon as possible to avoid perforation and worsening of inflammation. Delayed treatment can even lead to diffuse peritonitis, septic shock, and other life-threatening conditions. For patients with more severe appendicitis, the sooner the surgery is performed, the better, and there is no doubt about this. However, for uncomplicated acute appendicitis with stable conditions, there is still a lack of high-quality evidence regarding the specific time window for “early surgery”—whether it should be within 8 h or within 24 h, especially in the pediatric population. A multicenter randomized controlled trial (RCT) named PERFECT confirmed that for adults with suspected uncomplicated acute appendicitis, scheduling surgery within 24 h is not inferior to surgery within 8 h and does not increase the risk of perforation (1) There is a lack of relevant reports regarding whether this conclusion applies to pediatric patients. The pathophysiological characteristics of appendicitis in children significantly differ from those in adults, and arranging surgical resources for children is more challenging. Currently, there is still a lack of prospective or high-quality retrospective studies on the impact of preoperative delay in pediatric appendicitis. Therefore, this study aims to evaluate the impact of preoperative delay (<8 h vs. < 24 h) on the perforation rate and complications in children with Uncomplicated acute appendicitis undergoing LA at our hospital, through a single-center retrospective cohort analysis, in order to provide evidence for optimizing emergency surgical management strategies for pediatric appendicitis.

## 2 Methods

### 2.1 General information

**Figure 1:**
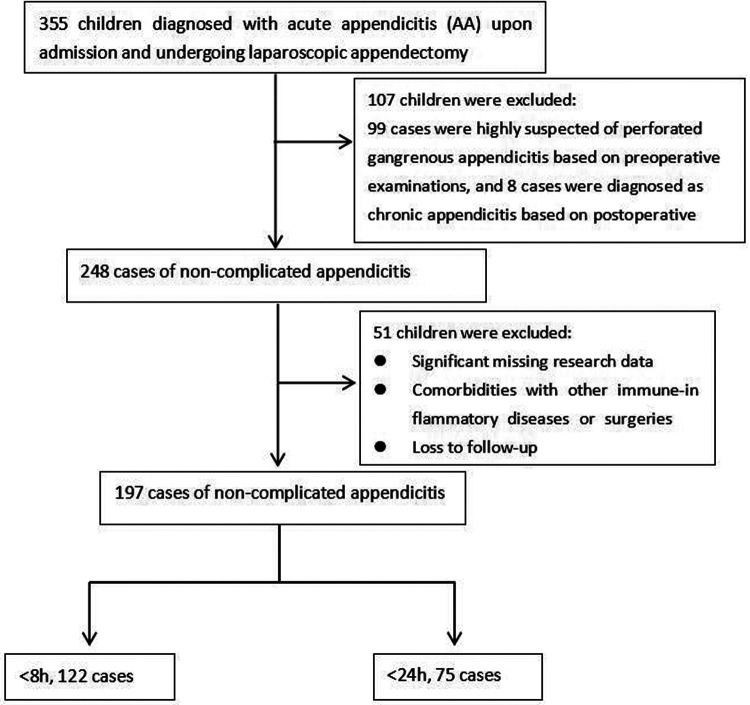
Flowchart of the enrolled children in the study.

This study is a single-center retrospective cohort study. Clinical data of children diagnosed with uncomplicated acute appendicitis (UAA) and undergoing laparoscopic appendectomy (LA) at the Department of General Surgery, Anhui Provincial Children's Hospital, between January 1, 2025, and December 31, 2025, were retrospectively collected. The diagnostic criteria for UAA were as follows: mild to moderate swelling of the appendix, serosal congestion, intra-luminal fluid accumulation, fibrous exudate on the surface, edema in all tissue layers under the microscope, neutrophil infiltration, and no necrosis or perforation, including simple and suppurative appendicitis without perforation. During the waiting period before surgery, all children received intravenous antibiotic treatment with third-generation cephalosporins for infection control and completed preoperative preparations on schedule. Informed consent was obtained from all children's guardians, and ethical approval was granted by the hospital's medical ethics committee. The child screening process for this study is shown in Figure 1.

### 2.2 Inclusion and exclusion criteria

#### 2.2.1 Inclusion criteria

(1) The admission diagnosis meets the criteria for pediatric UAA (Uncomplicated acute appendicitis), with preoperative examinations showing no signs of perforation or gangrene in the appendix; (2) The child's age is 18 years or younger; (3) All enrolled children underwent laparoscopic appendectomy (LA) without intraoperative conversion to open surgery, and the surgical procedure was standardized; (4) The clinical data included in the study is complete.

#### 2.2.2 Exclusion criteria

(1) Postoperative pathology indicating chronic appendicitis, acute exacerbation of chronic appendicitis, special types of appendicitis, appendiceal abscess, etc.; (2) History of other organ or tissue surgical operations; (3) Comorbidity with other inflammatory diseases such as respiratory or urinary tract infections; (4) Comorbidity with immune deficiencies, blood disorders, etc.

### 2.3 Study indicators and definitions

#### 2.3.1 The collected indicators include

1. Baseline data: age, sex, body mass index (BMI), ASA grade, vital signs upon admission, laboratory tests [white blood cell count (WCC), neutrophil count (NEUT), C-reactive protein (CRP)], and abdominal ultrasound characteristics (appendix diameter, wall thickness, fecaliths, omental echo enhancement);
2. Primary Outcome: The incidence of perforated appendicitis diagnosed intraoperatively based on the American Association for the Surgery of Trauma (AAST) grading system (grades 3–5);
3. Secondary Outcomes: operation time, postoperative length of stay, pathological results, postoperative complication rates (including surgical site infection, intra-abdominal abscess, intestinal obstruction), and total hospitalization costs.

#### 2.3.2 Preoperative delay definition

The interval between a definitive diagnosis (based on the time the surgical order is written) and the skin incision time (as recorded in the surgical log). Based on preoperative delay time, the children were divided into two groups: the control group (preoperative delay <8 h) and the observation group (preoperative delay <24 h).

#### 2.3.3 ASA grading definition

The ASA (American Society of Anesthesiologists) grading system is used to systematically assess the patient's overall health status and comorbidities before anesthesia. Its core value lies in providing a structured framework for anesthesiologists to assess perioperative risk by combining the patient's health status with key factors such as the type of surgery, patient frailty, and functional decline. See Table 1 for details (2).

**Table 1:** American society of anesthesiologists statement on ASA physical Status classification system.

ASA Classification	Definition	Pediatric Examples, including but not limited to:
ASA I	A healthy child	Healthy (no acute or chronic diseases), normal BMI percentile, age-appropriate development
ASA II	A child with mild systemic disease	Asymptomatic, non-cyanotic congenital heart disease, well-controlled arrhythmia, asthma without exacerbation, well-controlled epilepsy, non-insulin dependent diabetes, abnormal BMI percentile, mild/moderate OSA, cancer in remission, autism with mild restrictions, risk of malignant hyperthermia (family history or prior history)
ASA III	A child with severe systemic disease	Uncorrected stable congenital heart abnormalities, exacerbated asthma, poorly controlled epilepsy, insulin-dependent diabetes, morbid obesity, malnutrition, severe obstructive sleep apnea, tumor status, renal failure, muscular dystrophy, cystic fibrosis, organ transplant history, cranial malformation, symptomatic hydrocephalus, premature infant with PCA <60 weeks, severe autism, metabolic disorders, airway difficulties, long-term intravenous nutrition. Full-term infants <6 weeks of age
ASA IV	A child with severe systemic disease that is a constant threat to life	Symptomatic congenital heart disease, congestive heart failure, sequelae of preterm birth, acute hypoxic-ischemic encephalopathy, shock, sepsis, disseminated intravascular coagulation, automatic implanted cardiac defibrillator, mechanical ventilation dependence, endocrine disorders, severe trauma, severe respiratory distress, advanced cancer status
ASA V	A child whose life is in imminent danger and who will likely not survive without surgery	Severe trauma, intracranial hemorrhage with mass effect, requiring extracorporeal membrane oxygenation (ECMO), respiratory failure or cardiac arrest, malignant hypertension with hypertensive crisis, decompensated congestive heart failure, hepatic encephalopathy, ischemic bowel disease, or multiple organ/system dysfunction
ASA VI	A brain-dead child, organs removed for donation	

#### 2.3.4 AAST grading definition

The AAST (American Association for the Surgery of Trauma) appendicitis grading system is a standardized classification framework based on multidimensional assessments. By integrating clinical, imaging, surgical, and pathological criteria, the severity of the disease is quantified into levels 1 to 5. See Table 2 (3).

**Table 2:** AAST appendicitis grading system.

Grade	Description	Clinical Criteria	Imaging Criteria (CT)	Surgical Criteria	Pathological Criteria
1	Acute inflammation, intact	Pain, leukocytosis, right lower quadrant tenderness	Inflammation limited to the appendix, possibly with appendix dilation or incomplete contrast filling	Acute inflammation of the appendix, intact	Neutrophils present in the base of the crypt, submucosa and/or muscular layer
2	Gangrenous appendix, intact	Pain, leukocytosis, right lower quadrant tenderness	Necrosis of the appendix wall with no enhancement of contrast, possibly with intramural gas	Gangrenous appendix, intact	Mucosal and muscular layer digestion; Hematoxylin-eosin staining not identifiable
3	Perforated appendix, localized contamination	Pain, leukocytosis, right lower quadrant tenderness	As described above, with surrounding local fluid or contrast extravasation	As described above, with evidence of localized contamination	Gross perforation or focal muscular layer dissolution
4	Perforated appendix with periappendiceal cellulitis or abscess	Pain, leukocytosis, right lower quadrant tenderness, palpable mass	Regional soft tissue inflammation, cellulitis, or abscess	As described above, with abscess or cellulitis in the appendix region	Gross perforation
5	Perforated appendix with diffuse peritonitis	Diffuse peritonitis	Diffuse abdominal or pelvic inflammation, possibly with free abdominal fluid or gas	As described above, with widespread purulent contamination outside the appendix	Gross perforation

### 2.4 Follow-up

All children were routinely followed up after discharge (via telephone follow-up or outpatient visits). Postoperative complication data were collected by reviewing the electronic medical record system. The follow-up period was at least 30 days post-surgery.

### 2.5 Statistical methods

Statistical analysis was performed using SPSS 30.0 software. Normality and homogeneity of variance tests were conducted on all data. Normally distributed continuous data were expressed as mean ± standard deviation (SD), and inter-group comparisons were performed using the independent two-sample t-test. Skewed continuous data were expressed as median (P25, P75), and inter-group comparisons were performed using the Mann–Whitney *U*-test. For categorical data, the results were presented as *n* (%), and comparisons between groups were made using the *χ*² test or Fisher's exact test.

## 3 Results

### 3.1 Baseline data

A total of 355 children who underwent laparoscopic appendectomy were included in this study. Based on the inclusion and exclusion criteria, 197 children with suspected Uncomplicated acute appendicitis were included in the analysis. According to preoperative delay time, the children were divided into the control group (<8 h, *n* = 122) and the observation group (<24 h, *n* = 75). There were no statistically significant differences between the two groups in terms of gender, age, BMI, and ASA grading (*P* = 0.335, *P* = 0.985, *P* = 0.955, *P* = 0.387). Regarding appendicitis diagnostic indicators, there were no differences between the two groups in terms of white blood cell count, neutrophil count, C-reactive protein levels, appendix diameter measured by abdominal ultrasound, appendix wall thickness, fecalith detection rate, and surrounding omental echo enhancement signs (all *P* > 0.05). Clinical symptoms of acute appendicitis (including right lower quadrant tenderness, fever, vomiting, diarrhea) and the duration of illness (<24 h, 24–48 h, 48–72 h) also showed no statistical differences (*P* > 0.05). See Table 3.

**Table 3:** Comparison of baseline data between the Two groups.

Variable	Control Group, <8 h (*n* = 122)	Observation Group, <24 h (*n* = 75)	T/Z/*χ*2 Value	*P* Value
Gender				
Male	78 (63.9%)	43 (57.3%)	0.854	0.335
Female	44 (36.1%)	32 (42.7%)		
Age (years)	8 (6,10)	9 (6.5,11)	−0.019	0.985
BMI(kg/m^2^)	16.4 (14.5,17.8)	15.9 (15.2,17.4)	0.056	0.955
ASA score			1.897	0.387
1	87 (71.3%)	60 (80.0%)	NA	NA
2	32 (26.2%)	14 (18.7%)	NA	NA
3	3 (2.5%)	1 (1.3%)	NA	NA
4	0	0	NA	NA
WCC(×10⁹/L)	13.21 (12.79,13.71)	13.44 (12.84,14.44)	−0.457	0.646
NEUT(×10⁹/L)	11.78 (7.61,13.96)	10.87 (6.34,13.74)	0.314	0.754
CRP(mg/L)	9.93 (2.61,42.76)	9.35 (1.59,35.68)	1.340	0.180
Abdominal Ultrasound				
Appendix Diameter (mm)	7.29 ± 1.89	6.91 ± 1.04	1.598	0.112
Appendix Wall Thickness (mm)	2.14 ± 1.01	1.66 ± 0.55	1.419	0.158
Fecaliths	74 (60.6%)	39 (52.0%)	1.423	0.233
Omental Echo Enhancement	105 (86.1%)	61（81.3%）	0.784	0.376
Clinical Symptoms				
Right Lower Abdominal Tenderness	110 (90.2%)	68 (90.7%)	0.103	0.980
Fever (>37.5 °C)	56 (45.9%)	31 (41.3%)	0.393	0.531
Vomiting	47 (38.5%)	22 (29.3%)	1.724	0.189
Diarrhea	24 (19.7%)	15 (20.0%)	0.003	0.955
Duration of Illness			0.630	0.740
<24h	65 (53.3%)	41 (54.7%)	NA	NA
24–48h	53 (43.4%)	30 (40.0%)	NA	NA
48–72h	4(3.3%)	4(5.3%)	NA	NA

*P* < 0.05 indicates statistical significance.

### 3.2 Primary and secondary outcome indicators

In terms of the primary outcome, there was no significant difference in the incidence of AAST 3–5 grade perforated appendicitis between the observation group (<24 h) and the control group (<8 h) (9.3% vs. 7.4%, *χ*² = 0.238, *p* = 0.626). Regarding the secondary outcome indicators, there were no significant differences between the two groups in terms of surgical time, postoperative length of stay, appendix perforation based on pathological diagnosis, the incidence of postoperative complications, and total hospitalization costs (all *p* > 0.05). As the basis for study grouping, the preoperative delay time in the observation group was significantly longer than that in the control group [(14.4 ± 2.46) hours vs. (6.37 ± 2.30) hours, t = 23.17, *p* < 0.001], which was in line with the study design expectations. See Table 4.

**Table 4:** Comparison of primary and secondary outcomes between the Two groups.

Outcome	Control Group, <8 h (*n* = 122)	Observation Group, <24 h (*n* = 75)	*T/Z/χ² Value*	*P Value*
Primary Outcome				
Perforated Appendicitis (AAST 3–5)			0.238	0.626
AAST <3	113 (92.6%)	68 (90.7%)		
AAST 3–5	9 (7.4%)	7 (9.3%)		
Secondary Outcomes				
Surgical Time (mins)	39.5 (28.1, 45.3)	40.0 (31.5, 49.1)	−0.201	0.841
Length of Hospital Stay (days)	5.3 (4.1, 10.7)	5.5 (4.4, 9.6)	−0.314	0.754
Pathological Results			0.067	0.796
Non-perforated Appendicitis	101 (82.8%)	61 (81.3%)		
Perforated Appendicitis	21 (17.2%)	14 (18.7%)		
Preoperative Delay Time (h)	6.37 ± 2.30	14.4 ± 2.46	23.17	<0.001*
Postoperative Complications (cases)				
Surgical Site Infection	19 (15.6%)	7 (9.3%)	1.579	0.209
Intra-abdominal Abscess	25 (20.5%)	10 (13.3%)	1.629	0.202
Intestinal Obstruction	4 (3.3%)	2 (2.7%)	0.059	0.808
Treatment Cost (RMB)	11,947.38 ± 2,200.96	12,259.02 ± 2,328.42	0.944	0.346

*P < 0.05 indicates a statistically significant difference.

### 3.3 Subgroup analysis of children with appendiceal fecalith

Appendiceal fecalith is generally considered a potential risk factor for appendiceal perforation. In this study, a subgroup of children with preoperative imaging suggesting the presence of appendiceal fecaliths (74 cases in the control group, 39 cases in the observation group) was compared. The results showed that the AAST perforation rates for the control and observation groups were 7 (9.46%) and 6 (15.38%), respectively, but with *P* = 0.348, there was no significant difference. However, significant differences were found between the two groups in terms of surgical time [38.7 (25.1, 44.6) vs. 41.0 (33.4, 52.5)] minutes, pathological results (12.16% vs. 28.21%), and preoperative delay time (6.21 ± 2.00 vs. 13.89 ± 2.23) hours (*P* = 0.045, *P* = 0.034, *P* < 0.001). These results suggest that, although there was no difference in the AAST perforation rate, the final pathological results showed a difference, indicating that fecalith is an important factor influencing the risk of appendiceal perforation. See Table 5.

**Table 5:** Comparison of primary and secondary outcomes between the Two groups of children with positive appendiceal fecalith.

Outcome	Control Group, <8 h (*n* = 74)	Observation Group, <24 h (*n* = 39)	T/Z/χ² Value	*P* Value
Primary Outcome				
Perforated Appendicitis (AAST 3–5)			0.881	0.348
AAST <3	67 (90.5%)	33 (84.6%)		
AAST 3–5	7 (9.5%)	6 (15.4%)		
Secondary Outcomes				
Surgical Time (mins)	38.7 (25.1, 44.6)	41.0 (33.4, 52.5)	−2.100	0.045*
Length of Hospital Stay (days)	5.4 (4.5, 12.7)	6.5 (4.5, 11.3)	−0.540	0.592
Pathological Results			4.513	0.034*
Non-perforated Appendicitis	65 (87.8%)	28 (71.8%)		
Perforated Appendicitis	9 (12.2%)	11 (28.2%)		
Preoperative Delay Time (h)	6.21 ± 2.00	13.89 ± 2.23	18.65	<0.001*
Postoperative Complications (cases)				
Surgical Site Infection	9 (12.2%)	4 (10.3%)	0.091	0.763
Intra-abdominal Abscess	11 (14.9%)	6 (15.4%)	0.005	0.941
Intestinal Obstruction	2 (2.7%)	2 (5.1%)	0.016	0.898
Treatment Cost (RMB)	11,836.26 ± 2,197.53	12,117.01 ± 2,425.17	0.623	0.535

*P < 0.05 indicates a statistically significant difference.

### 3.4 Follow-up results

All 197 children completed at least 30 days of postoperative follow-up. No new or additional medium- or long-term complications were observed during the follow-up, and no deaths occurred, indirectly confirming the safety of preoperative delay in Uncomplicated acute appendicitis.

## 4 Discussion

Acute appendicitis is the most common acute abdominal condition in pediatric surgery, and the timing of surgery has always been one of the core controversies in clinical decision-making (4). On the other hand, there is also controversy regarding the consequences of delayed appendectomy for acute appendicitis (5, 6). This study, through a single-center retrospective cohort analysis, explored whether delaying laparoscopic appendectomy (LA) to within 24 h for children with preoperative diagnosis of Uncomplicated acute appendicitis (UAA), compared to traditional emergency surgery within 8 h, would increase the risk of adverse perioperative outcomes. The main finding was that in the overall population, a preoperative delay of less than 24 h did not significantly increase the incidence of intraoperative perforation (AAST 3–5), and there were no differences between the two groups in terms of surgical time, length of hospital stay, complications, or treatment costs. However, in the subgroup of children with appendiceal fecalith, delayed surgery was associated with a higher pathological perforation rate, suggesting that fecalith is an important risk factor.

In this study, no statistical differences were found between the control and observation groups in terms of gender, age, BMI, ASA score, preoperative blood parameters (WCC, NEUT, CRP), ultrasound parameters (appendix diameter, width, presence of fecalith, omental echo enhancement), clinical symptoms (right lower abdominal pain, fever, vomiting, diarrhea), or duration of illness (<24 h, 24–48 h, 48–72 h) (*P* > 0.05), indicating that the study design was balanced and comparable. Further analysis of the primary outcome for children with UAA showed that there were no statistically significant differences in the intraoperative AAST perforation rates between the control group (<8 h) and the observation group (<24 h) (7.4% vs. 9.3%, respectively). Preoperative delay did not increase the perforation rate, which is consistent with the results of Agnesi et al. (7), who found through univariate and multivariate regression analysis that hospital delay, as both a continuous and categorical variable, was not associated with postoperative appendicitis severity. Even when the population was divided into low, medium, and high-risk groups based on the Alvarado score, surgery time was not correlated with the severity of appendicitis in any risk group, with *P* values of 0.78, 0.28, and 0.24, respectively.

Additionally, preoperative delay did not increase perioperative adverse outcomes in acute appendicitis. UAA includes simple appendicitis and suppurative non-perforated appendicitis, where inflammation is more localized, and the seromuscular barrier of the appendix is usually intact. With effective antibiotic therapy, it can achieve a cure. Generally, the duration of illness is related to the type of appendicitis. Clinically, the longer the duration of appendicitis, the greater the likelihood of perforation, intra-abdominal infection, and peritonitis, which can be confirmed in clinical practice. Theoretically, preoperative delay, which increases the duration of illness, may also lead to these consequences. However, some studies have reported that the mechanisms of inflammation in acute appendicitis with different severity levels are not the same, and time is not a critical factor for appendiceal perforation. Carvalho et al. (8) reported that gangrenous appendicitis is related to Th1 inflammatory response, while suppurative appendicitis is related to Th2 cell response, with cytokines such as IL-4, IL-5, IL-9, IL-13 involved. A prospective study analyzing IL-4, IL-9, IL-13, and total IgE concentrations in 138 patients under 15 years old found that serum concentrations of IL-4 and IL-9 did not affect the risk of complicated appendicitis, but high levels of IL-13 were associated with an increased risk of complicated appendicitis (9). Type I hypersensitivity also plays a role in the occurrence and progression of acute appendicitis (10). Li et al. (11) reported that a shorter preoperative delay for appendectomy does not increase the risk of complicated appendicitis but is slightly associated with an increased risk of surgical site infection. To avoid additional morbidity, timely surgical intervention is recommended for faster recovery, which is consistent with the findings of this study, where a short preoperative delay in UAA did not lead to worse outcomes.

In the secondary outcome analysis, the two groups of children showed high consistency. We compared surgical time, length of hospital stay, hospitalization costs, pathological perforation rates, and postoperative complications, and the results showed no significant statistical differences (*P* > 0.05), confirming that preoperative delay up to 24 h for UAA is feasible. Despite the significantly longer preoperative waiting time in the observation group (14.4 ± 2.46 vs. 6.37 ± 2.30) hours, the incidence of postoperative complications did not increase, which somewhat reflects the safety of delayed surgery for UAA under effective antibiotic coverage (12). This is consistent with the study by Collins CM et al., which concluded that lower-grade appendicitis corresponds to more predictable and lower medical resource demands (13).

Traditional beliefs suggest that delayed surgery is a risk factor for appendiceal perforation, but recent evidence suggests that “pre-hospital delay,” the time from symptom onset to seeking medical attention, may be a more critical influencing factor. The impact of in-hospital, controllable waiting time (especially within 24 h) on disease progression is limited. Elliott BM's research reported that complicated appendicitis was associated with prolonged pre-hospital delay (47.8 h vs. 20.1 h, *P* < 0.001), but not with preoperative delay time (11.3 h vs. 13.3 h, *P* = 0.96) (14). This study found that the occurrence of perforation was not directly associated with preoperative delay, and there were no statistical differences in perioperative outcomes between the two groups. However, subgroup analysis revealed a difference. In children with appendiceal fecalith detected by preoperative ultrasound, there was no statistical difference in the AAST perforation rate between the two groups (*χ*² = 0.881, *P* = 0.348), but the pathological perforation rate in the observation group was significantly higher than in the control group (28.21% vs. 12.16%, *P* = 0.034), and the surgical time was also longer [38.7 (25.1, 44.6) vs. 41.0 (33.4, 52.5)] minutes. The discrepancy between the AAST and pathological results may be due to the limitations of intraoperative assessment or differences among graders. However, the pathological diagnosis, as the gold standard, is more noteworthy. Appendiceal fecalith significantly accelerates the progression of inflammation to gangrene and perforation by causing persistent lumen obstruction, increased internal pressure, and local ischemia (15). Tang et al. confirmed that appendiceal fecalith is an independent risk factor for UAA perforation, which is consistent with the evidence of this study (16). The results of this study suggest that even with preoperative evaluation as “non-complicated,” the presence of fecalith may cause the appendix to “silently” progress to microscopic or localized perforation during an extended waiting period. This explains why the difficulty of surgery in this subgroup increases (surgical time is longer). Therefore, appendiceal fecalith is an important risk factor for delayed surgery in UAA patients. For these patients, a more proactive surgical approach (such as performing surgery within 8 h if possible) may benefit them to avoid potential pathological progression.

During the 30-day follow-up period, the complication rates were low in both groups, with no severe complications or deaths, further confirming the safety and reliability of preoperative delay of <24 h in pediatric UAA. However, this study also has several limitations. First, as a retrospective cohort study, it is subject to potential selection bias and confounding factors. For example, data from 158 children were excluded from the analysis. Although most exclusions were due to incomplete preoperative imaging or laboratory data, missing postoperative pathology results, or loss to follow-up, these exclusions may still have introduced selection bias. In addition, the assessment of prehospital delay (defined as the interval from symptom onset to presentation/diagnosis) primarily relied on retrospectively collected caregiver-reported histories, which may have been subject to recall bias. Second, the definition of preoperative delay in this study also has certain limitations. In the present study, preoperative delay was defined as the interval from confirmed diagnosis (or surgical order placement) to skin incision, rather than including the “prehospital delay” from symptom onset to diagnosis as the primary exposure variable. This definition differs from those commonly used in previous studies, which often define delay as the interval from patient presentation or hospital admission to surgery, and therefore may limit direct comparisons between our findings and previously published reports. Future studies should more precisely quantify prehospital delay and further investigate its interaction with in-hospital preoperative delay in order to clarify their independent and combined effects on the risk of appendiceal perforation. Third, validated clinical scoring systems, such as the Alvarado Score or Pediatric Appendicitis Score, were not used to stratify disease severity in the enrolled children. This may have introduced indication bias, whereby children with more severe disease were prioritized for earlier surgery (shorter preoperative delay), whereas those with milder disease may have experienced delayed surgery (longer preoperative delay), potentially confounding the assessment of the true impact of preoperative delay.

In conclusion, this study confirms that for children with preoperative evaluation of suspected Uncomplicated acute appendicitis, deliberately delaying laparoscopic appendectomy to within 24 h does not significantly increase the incidence of intraoperative perforation and postoperative complications. This provides evidence for daytime surgery scheduling and optimizing nighttime emergency resources. However, for children with preoperative detection of appendiceal fecalith, delayed surgery may increase the risk of perforation, and a more proactive surgical strategy is recommended for this group of patients.

## Data Availability

The original contributions presented in the study are included in the article/Supplementary Material, further inquiries can be directed to the corresponding author/s.
